# Assessment of supportive care needs among cervical cancer patients under treatment in Nepal: a cross-sectional study

**DOI:** 10.1186/s12905-023-02484-z

**Published:** 2023-08-03

**Authors:** Kamala Dhakal, Panpan Wang, Joanes Faustine Mboineki, Mikiyas Amare Getu, Changying Chen

**Affiliations:** 1 Nursing Department, The first affiliated hospital of Zhengzhou University, Jianshe Dong Lu, Henan Province, Zhengzhou, Henan 450000 China; 2 School of Nursing and Health, Zhengzhou University, Zhengzhou, Henan China; 3 Maharjgunj Nursing Campus, Maharajgunj, Kathmandu Nepal; 4 Institute for Hospital Management of Henan, Jianshe Dong Lu, Henan Province, Zhengzhou, Henan 450000 China

**Keywords:** Cervical cancer, Supportive care, Supportive care needs, Assessment

## Abstract

**Backgrounds:**

The perceived supportive care needs (SCNs) of cancer patients are essential components of a care program. The first step in planning and intervening for supportive care is the proper identification of the SCNs of cancer patients. Cervical cancer (CC) is the most prevalent cancer among Nepali women. The authors assess SCNs and their predictors among CC patients under treatment by using a validated Nepali version supportive care need survey short form (SCNS- SF 34 N).

**Methods:**

This descriptive cross-sectional study was conducted in 5 cancer treatment hospitals in Nepal. A culturally adapted and psychometrically validated Nepali version SCNS –SF- 34 N was completed by a convenience sample of 218 CC patients. Data were analyzed by using descriptive (frequency, percentage, mean, median) and inferential (Chi-square *P*-value and binary logistic regression analysis) statistics.

**Results:**

The study showed that 99% of the respondents were in need of some level (low, moderate, high) of supportive care. The psychological domain, physical daily living, health system information, patient care support and sexuality domain ranked from first to fifth for SCNs with mean and standard deviations 70.29 ± 18.84, 63.25 ± 23.15, 57.90 ± 21.11, 56.46 ± 21.92 and 46.06 ± 34.16, respectively. Binary logistic regression found causal association between SCNs and variables “occupation (p-value = 0.007), and type of hospital (p-value = 0.000)” at a 95% confidence level.

**Conclusion:**

Nepali CC patients perceive and experience many unmet SCNs, with psychological SCNs being the first priority. It is essential that the SCNs of patients may need to be known by their close family members, care providers, CC related program. so that they can offer intervention as per patients’ needs.

## Background

Globally, cervical cancer (CC) is ranked the fourth most commonly diagnosed cancer [1, 2] and the fourth foremost cause of cancer death in women, with an estimated 604,000 new cases and 342,000 deaths in 2020 [1]. In women of low and middle-income countries (LMICs), it is the second most common cancer. According to worldwide data of 2018, 51% of the 570,000 new CC cases occurred in women living in LMICs (88%) compared to those living in middle-income countries [3]. It is the fourth most common cancer and near about 5,70,000 and 3,11,000 new cases and death cases, respectively [4]. In women of the South East Asia region, CC is the second leading cancer and it is the main cause of cancer-related death in LMICs like Nepal [5]. In the worldwide scenario, from 1990 to 2019, the trend of CC decreased, especially in incidence, death, and disability-adjusted life years (DALYs) with estimated annual percentage change (EAPCs) of − 0.38 (95% confidence interval [CI]: − 0.41 to − 0.34) for incidence, − 0.93 (95%CI: − 0.98 to − 0.88) for death, and − 0.95 (95 CI%: − 1.00 to − 0.90) for disability. Decreasing trends were found in most sociodemographic index (SDI) areas and geographic regions, particularly death and DALYs in Central Latin America with EAPCs of − 2.61 (95% CI: − 2.76 to − 2.46) and − 2.48 (95% CI: − 2.63 to − 2.32). On the other hand, there was a noticeable increasing trend in the incidence of CC in East Asia (EAPC = 1.33; 95% CI: 1.12 to 1.55) [6].

In Nepal, CC is the first common cancer among women and it commonly occurs between 15 and 44 years of age, with 2,332 new cases and 1,367 death cases every year [7]. The incidence of CC in National Tertiary Cancer Hospital (B.P. Koirala Memorial Cancer Hospital) in Nepal was relatively constant, 2012–2017 with topmost 605 cases in the year 2016 and lowermost 459 cases in the year 2014. The most common age at diagnosis was 45–59 years (44.6%) followed by 60–74 years (29.4%) and 30–44 years (21.1%) [8]. According to a study carried out in Nepal among CC patients, 75% of the respondents were in equals to and greater than stage II of the disease and the rest (25%) of the respondents were in lesser than stage II of the disease at the time of diagnosis [9] in 2021.

CC is a type of cancer that occurs in the cells of the cervix: the lower part that connects the uterus with the vagina. Human papillomavirus infection is the main cause of CC along with many risk factors like early marriage, early intercourse, having multiple sexual partners, smoking etc. It starts with no symptoms, but later it presents with pain during sexual intercourse and vaginal bleeding. The treatment consists of a combination of modalities such as surgery, chemotherapy, and radiotherapy [10].

In comparison with other gynecological cancer patients, CC patients face manifold problems such as physical, psychological and social distress, spiritual suffering, fatigue, irritability, memory loss, decreased energy level, recurring pain, worse emotional distress and poor quality of life [11]. CC patients commonly experience anxiety and feel that they are going to die in a devastating condition [12]. According to a hospital-based cross-sectional study in Ethiopia, 79.7% of CC patients experienced anxiety and 47% of CC patients felt depression [13]. With the help of supportive care (SC), patients and family members can manage these disease-related problems in a comprehensive and holistic manner during the disease course [14, 15].

SC starts immediately after the diagnosis; it is a mixture of health services and related activities which help patients and their families in all phases of the disease in a balanced way: diagnostic, treatment, follow-up, and palliative phases of cancer [16]. It is essential to measure and identify the SCNs of CC patients at first for appropriate planning of necessary SC in all phases of cancer disease, including diagnosis and treatment [16, 17].

The supportive care need survey assesses the category and grade of perceived SCNs of cancer patients covering five domains: psychological, health system information, physical daily living, patient care support, and sexuality. It is one of the most complete, reliable and valid SCNs assessment tools for cancer patients [18, 19].

The assessment of SCNs helps to identify individual living needs perceived and expressed by CC patients, which include physical, psychological, emotional, social, spiritual, and practical needs. After the identification of SCNs, healthcare providers can provide targeted SC. SC helps CC patients and their families to cope with cancer symptoms and side-effects of treatments in all phases of cancer: before diagnosis, during diagnostic procedures, treatment phase, and recovering phase of illness or even at the phase of death [20]. SC can provide valuable benefits to patients and their families to handle and cope with the crisis caused by CC effectively [21].

For the assessment of SCNs, several studies have been conducted with various types of cancer patient populations worldwide, most of which are limited to western countries [14, 16, 17, 22–24]. Only a small number of studies have been conducted in Asian countries among cancer patients for the assessment of SCNs [25–28]. The results of these studies conducted in different countries show that there prevail differences in SCNs of patients with different cancer diagnoses [14]. Fatigue, pain, and nausea are the most commonly reported SCNs in gynecological cancer patients, such patients often suffer from psychological problems such as depression, anxiety, and nervousness and CC is one of the most common gynecological cancers [25, 29, 30]. CC patients, in comparison with other gynecological patients, experience relatively a higher level of anxiety and fear that they are going to die in a devastating condition. This type of psychological problem may also bring about social problems. The most common social problems for CC patients include dependency, role dysfunction, loss of productivity and social isolation [12, 31]. Compared to other cancer patients, women with CC show strong concern about sexuality/intimacy issues and information-seeking [32].

CC patients in Seoul shared that they were in need of sexuality and psychological SC to cope up with sexual dysfunction (4.83 ± 4.16) and moderate and severe depression (11.08 ± 5.06)) [33]. CC patients under the treatment in a cancer hospital in Zambia needed psychological supportive care: 80% of patients reported depressive symptoms, 78% moderate, 18% mild and 4% severe [34]. CC patients in Western Kenya experienced several financial challenges and they were in need of financial SC : costs of medication 87%, cost of travel 84% and cost of diagnostic tests 75% [35]. The highest priority of SCNs was identified in the health system information-related domain among CC patients in Thailand [36]. The most common complaint among CC patients was the loss of concentration (85.0%) and symptom management strategies (55.0%), and counseling services (40.0%) were identified as common SCNs in Mainland China [26].

The SCNs of CC patients can be affected by age, marital status, socioeconomic status, dietary habits, site of cancer, and chemotherapeutic agents are reported to be influencing factors on patterns of adverse drug reaction due to cancer chemotherapy and their management in the hospitalized patient [37]. Age, gender, education, ethnicity, occupation and marital status were the influencing factors on the perception of side effects of chemotherapy among cancer patients [38]. Likewise, age, education, remoteness of place of diagnosis and socioeconomic status were the influencing factors associated with late diagnosis of CC in Nepal [39], whereas age, sex, marital status, religion, ethnicity, education, occupation, type of family and economic status were the key factors influencing the quality of lives of patients undergoing cancer treatment [40].

Regarding the SC program for CC patients in Nepal, the population-based cancer registry (PBCR) was initiated by Nepal Health Research Council, a national body of the Government of Nepal, in January 2018. This program is running in 8 districts of Nepal (Kathmandu, Lalitpur, Bhaktapur, Siraha, Saptari, Dhanusa, Mahottari and Rukum). PBCR covers approximately 20% of Nepal’s total population and is expanding [41]. According to Family Health Division, 2010, CC screening (using visual inspection with an acetic acid approach) and prevention programs are running in Nepal complying with the national guidelines developed by the Division of Family Health within the Department of Health in 2010. It targeted to cover 50% population of 30–60 years of age within 5 years, as cited by a community-based intervention study in Nepal [42]. According to a hospital-based cross-sectional study the rate of CC screening in Nepal is poor, only 18.8% of the respondents had done CC screening at once and other remaining respondents had never done CC screening [43].

At present, there exists no national-level cancer control program in Nepal. Only two public-funded cancer centers are available in Nepal, both are located in central Nepal [44]. Additionally, some emerging private hospitals are offering cancer care [45]. Palliative care in hospitals started in Bir Hospital in 1991[46]. After that, Nepal initiated a national strategy for providing appropriate palliative care in all parts of the country only in 2017[44]. At present, most cancer-specific hospitals are treating patients in their own palliative care units. There is also the availability of three oncology palliative centers along with a palliative care unit in the hospital [46, 47]. Palliative care helps to optimize the comfort, function, and social support for patients and their families when a cure is not possible, whereas SC helps to optimize the comfort, function, and social support for patients and their families at all stages of the illness. CC patients in Nepal can get care to optimize comfort, function, and social support in cancer hospitals and palliative care units [20].

The extensive review of the literature on this issue shows that there has been no study in Nepal reporting the assessment of the SCNs of cancer or CC patients. This claim was further substantiated by our search of PubMed database on 1st December 2021 by using 5 search terms: cervical cancer, cancer, supportive care, supportive care needs and assessment.

Out of 286 records, only 16 articles were found to be related to SCNs and 5 of them were review articles not specific to Nepal, and 11 articles reported the studies conducted in other countries, namely in Indonesia, Netherlands, Denmark, Spain, Australia, Northern Ireland, Amsterdam, Canada, Japan, and the USA. Considering this research gap, the researchers aimed to assess SCNs and their predictors among CC patients in Nepal.

## Methodology

### Aim

This study aimed to identify SCNs and their predictors among CC patients under treatment in Nepal. This study was formally approved by the School of Nursing & Health, Zhengzhou University (ZZU IRB 2019-028), Henan China and Nepal Health Research Council (Ref. No.1706).

### Design

A descriptive cross-sectional study design [14, 16, 17, 22–25, 27, 28, 30] was used to find out the SCNs and their predictors among CC patients in Nepal.

### Study setting

This study was carried out in 5 cancer-specific hospitals in Nepal, namely Bhaktapur Cancer Hospital (BCH), Bhaktapur, Nepal and Bisweswar Prasad Koirala Memorial Cancer Hospital(BPKMCH), Bharatpur, Chitwan, Nepal Cancer Hospital & Research Center (NCHRC); Harisiddhi, National Hospital &Cancer Center (NHCC); Jawalakhel, Kathmandu Cancer Center; Tathali, Nepal from October 2020 to March 2021.

In Nepal, there are 38 hospitals, government and private, which provide general and special care and treatment to patients. Among them, 6 hospitals and 2 hospice centers (Bhaktapur Cancer Hospital, Kathmandu Cancer Centre, Nepal Cancer Hospital and Research Centre, National Cancer Hospital, Thankot Hospice, Hospice Nepal, B.P Koirala Memorial Cancer Hospital and Sushil Koirala Prakhar Cancer Hospital) provide cancer-specific care and treatment. Only one out of eight cancer-related centers is a fully government-owned hospital; one hospital is in a public-private partnership, whereas four private hospitals and the rest two are privately-run hospice centers. Four out of the six cancer-specific hospitals are situated in Kathmandu Valley (Bhaktapur Cancer Hospital, Kathmandu Cancer Centre, Nepal Cancer Hospital and Research Centre, National Cancer Hospital,) and the other two (B.P Koirala Memorial Cancer Hospital and Sushil Koirala Prakhar Cancer Hospital) are located out of Kathmandu Valley [41]. Only cancer-specific hospitals were included in this study. Five out of 6 cancer-specific hospitals were included in the current study. Sushil Koirala Prakhar Cancer Hospital, located in the far western region of Nepal, was not included in the study due to a feasibility issue. Likewise, hospice care centers were not included in the study, since their service concentrated only on compassionate care for people in the last phases of the incurable disease.

### Characteristics of participants

The inclusion criteria were patients diagnosed with CC, undergoing cancer therapy, physically and mentally able to complete the questionnaire (can speak and give meaningful responses) [25, 26] and understanding the Nepali language.

#### Operational definitions

### Economic status

Economic status was operationally defined by dividing respondents into three categories as per the respondents’ perceived verbal answer about their income: their income is adequate or enough to sustain for one year; their income is not adequate or enough to sustain for one year; and their income is adequate with extra saving.

### Relationship status

Relationship status was measured in terms of whether the husband and wife were living together or not from the date of diagnosis of the disease to the date of data collection. It was measured by living together, *yes*, and not living together, *no*.

### Currently sexually active

Current sexual activity was measured by whether or not respondents participated in sexual activity from the date of diagnosis to the date of data collection. It was measured by *yes* or *no*.

### Patients with no need for supportive care

The respondent who answered *1—no need, not applicable; and or 2—no need, satisfied;* in all 34 items of Supportive Care Needs Survey Short Form 34 Nepali (SCNS-SF 34 N) came under patients with no need for supportive care.

### Patients with some need for supportive care

The respondents who answered *3—low need; and or 4—moderate need; and or 5—high need* in any one of the 34 items of SCNS-SF 34 N came under patients with some need for supportive care.

### Sample size

The sample size was calculated by using G power calculation [48], and the assessment of supportive care needs was conducted with a sample of 218 cc patients. While calculating the sample size, the following parameters were used: Input Parameters = Effect size f^2^ = 0.15, α err prob = 0.05, Power (1-β err prob) = 0.95, Number of tested Predictors = 19, Total number of Predictors = 34.

Total sample size = 218.

### Sampling

The convenience sampling technique was used for the selection of 218 CC patients [16, 17, 25, 49] from the selected hospitals to assess the respondents’ supportive care needs. During the data collection period of 5 months, the principal researcher and the research assistants visited each hospital on a particular day once a week. The CC patients of each particular day from a particular hospital were taken as a sample, and at the end of each week, the total sample from all hospitals was calculated. The enrollment was stopped after getting the required number of participants as a sample from the five hospitals mentioned above.

### Data collection tools and procedures

SCNS SF-34 was used as an instrument for data collection. It consisted of 34 items across 5 factors of analytically-originated domains which are the same as the longer version: psychological (10 items), health system information (11 items), physical daily living (5 items), patient care support (5 items), and sexuality (3 items). The tool gives information regarding present needs and degrees of support needed for the patient in the previous month due to cancer (1—no need, not applicable; 2—no need, satisfied; 3—low need; 4—moderate need; 5—high need). A high score in the tool indicates that the level of perceived supportive care needs is high [50].

Translation and cultural validation of SCNS SF-34 was carried out by following Beatons’ Guidelines [51] and content validity assessment [52], followed by the confirmation of the psychometric properties of the Nepali version of SCNS- SF-34 N through explanatory factor analysis [49, 53, 54]. Pretesting was conducted in specified cancer hospitals with 10% of the sample and the pretested samples were not included in this study. The SCNs questionnaire was assessed by using the psychometrically validated Nepali version- SCNS-SF 34 N (Item Content validity index (I-CVI) > 0.78, Scale Content validity index (S-CVI)- 0.91, Cronbach’s alpha coefficients ranging from 0.789 to 0.929 for all five domains, and 0.887 for the whole scale, no floor and ceiling effect with 65.48% of the total variance in five domain of questionnaire). Apart from SCNS questionnaires, only demographic-related information was included in the study.

The purpose of the study was explained to the participants, informed written consent was taken, and the questionnaire was provided for completion [17, 49]. For participants who were unable to complete the questionnaire due to poor reading and writing abilities, the researcher and research assistants (Nursing staff, who passed Bachelor’s degree in nursing) helped them via a short interview. Brief training was given to the research assistants on how to collect data before going to the field for data collection [17, 26].

### Data analysis

The data were analyzed by using the Statistical Package for Social Science(SPSS) version 20(IBM, NY, USA). Descriptive statistics (frequency, percentage, mean, median and standard deviation) [29, 55] for identification of demographic data and CC-related information, and unmet and met SCNs of participants and inferential statistics (Chi-square *P*-value and binary logistic regression analysis) [56] were used to predict the factors affecting SCNs among CC patients.

## Results

### Demographics and disease-related characteristics of respondents

The mean age and standard deviation of the respondents were 53.51 and ± 12.48, respectively. More than three-fourths, i.e. 80.2%, of the respondents were illiterate. In the same way, 49.1% and 32.6% of the respondents were in the 2nd and 3rd stages of the disease respectively. Moreover, 67.9% of the respondents were getting the treatment through radiation and chemotherapy modalities. (Refer to Table 1)

**Table 1:** Demographics and disease-related characteristics of respondents (n = 218)

Variables	Frequency	Percent(%)
**Age in years**		
<= 45.00	61	28.0
46.00–65.00	115	52.8
66.00 +	42	19.3
**Mean age, SD**	53.51 ± 12.48	
**Religion**		
Hindu	184	84.4
Buddhist	25	11.5
Christian	9	4.1
**Caste**		
Brahman	35	16.1
Chhettri	64	29.4
Newar	26	11.9
Madheshi	26	11.9
Dalit	14	6.4
Janjati	45	20.6
Aadibasi	8	3.6
**Education**		
Illiterate	175	80.2
Literate	43	19.7
**Marital status**		
Married	164	75.2
Single/Widow	51	23.4
Unmarried	3	1.4
**Residence**		
Urban	79	36.2
Rural	139	63.8
**Occupation**		
Agriculture	99	45.4
Housewife	102	46.8
Job	9	4.1
Business	8	3.7
**Economic status**		
Enough to sustain for one year	149	68.3
Not enough to sustain for one year	53	24.3
Extra saving	16	7.3
**Dietary status**		
Vegetarian	43	19.7
Non-vegetarian	175	80.3
**Family type**		
Nuclear	80	36.7
Joint	122	56.0
Extended	16	7.3
**Past exp. cervix disease self**		
No	190	87.2
Yes	28	12.8
**Name of problem (n = 28)**		
Watery discharge	17	60.7
Bleeding	11	39.2
**Relationship status**		
Not living together	83	38.1
Living together	135	61.9
**Currently sexually active**		
No	155	71.1
Yes	63	28.9
**Stage of disease**		
Stage I	26	11.9
Stage II	107	49.1
Stage III	71	32.6
Stage IV	14	6.4
**Treatment modalities**		
Operation	3	1.4
Radiation	32	14.7
Operation + Chemotherapy	6	2.8
Operation + Radiation	6	2.8
Radiation + Chemotherapy	148	67.9
Operation + Chemotherapy + Radiation	23	10.6
**Duration of disease**		
< 1.00 Year	201	92.2
1.00–2 Year	12	5.5
2.00 + Year	5	2.3
Median duration (Q1,Q3)	3.96(1.92,5.19) months	

Frequency (n) and percentage (%) were used for the analysis of participants’ demographic and disease-related characteristics.

### Patients’ supportive care needs

Worries about those close to them (94%), feelings about death and dying (92.2%), fear about cancer spreading (91.7%), and anxiety (91.7%) were four of the 12 most frequently reported items in the psychological domain of supportive care needs. Being informed about cancer that is under control or diminishing i.e., remission (78.9%) was the most frequently reported item under the health system information domain that contained 9 items. Among 5 items under the physical and daily living domain, lack of energy/tiredness/fatigue (88.5%) was the most frequently reported item for supportive care needs, whereas hospital staff attending promptly to their physical needs (73.9%) was the most frequently reported item under the 5-itemed patient and support domain. Likewise, among 3 items under the sexuality domain, the item concerning the changes in their sexual relationship (61.6%) was the most frequently reported one. Almost all (99.08%) of the respondents stated that they needed some form of supportive care (low, moderate, high).

The majority (i.e. 82.57%**)** of the respondents said that they t needed some level of supportive care in the physical and daily living domain. In a similar vein, 72.48% of the respondents reported needing some (low, moderate or high) level of supportive care in the patient care and support domain, and 61.01% of them were seeking some support in the sexuality domain. (Refer to Table 2)

**Table 2:** Item-wise assessment of supportive care needs (n = 218)

Domain & item	Unmet supportive care needs				
	Low	Moderate	High	Some need	No Need
**Psychological domain**					
Being treated like a person not just another case	43(19.7%)	65(29.8%)	35(16.1%)	143(65.6%)	75(34.4)
Worry that the results of treatment are beyond your control	45(20.6%)	72(33.0%)	79(36.2%)	196(89.8%)	22(10.2)
Keeping a positive outlook	56(25.7%)	84(38.5%)	53(24.3%)	193(88.5%)	25 (11.5)
Feelings about death and dying	54(24.8%)	85(39.0%)	62(28.4%)	201(92.2%)	17 (7.8)
Uncertainty about the future	45(20.6%)	74(33.9%)	71(32.6%)	190(87.1%)	28 (12.9)
Feelings of sadness	53(24.3%)	84(38.5%)	61(28.0%)	198(90.8%)	20 (9.2)
Fear about cancer spreading	38(17.4%)	74(33.9%)	88(40.4%)	200(91.7%)	18 (8.3)
Anxiety	53(24.3%)	91(41.7%)	56(25.7%)	200(91.7%)	18 (8.3)
Feeling down or depressed	64(29.4%)	78(35.8%)	52(23.9%)	194(89.1%)	24 (10.9)
Being treated in a hospital or clinic that is as physically pleasant as possible	78(35.8%)	55(25.2%)	22(10.1%)	155(71.1%)	63 (28.9)
Worries about those close to you	55(25.2%)	68(31.2%)	82(37.6%)	205(94%)	16 (6)
Learning to feel in control of your situation	64(29.4%)	91(41.7%)	28(12.8%)	183(83.9%)	35 (16.1)
**Psychological supportive care need**				**189(86.97)**	**29(13.03)**
**Health system information domain**					
Being adequately informed about the benefits and side effects of treatments before you choose to have them	52(23.9%)	67(30.7%)	38(17.4%)	157(72%)	61 (28)
Being informed about your test results as soon as feasible	52(23.9%)	67(30.7%)	33(15.1%)	152(69.7%)	66 (30.3)
Being informed about things you can do to help yourself to get well	57(26.1%)	71(32.6%)	33(15.1%)	161(73.8)	57 (26.2)
Being given information (written, diagrams, drawings) about aspects of managing your illness and side effects at home	54(24.8%)	53(24.3%)	58(26.6%)	165(75.7%)	53 (24.3)
Having one member of hospital staff with whom you can talk about all aspects of your condition, treatment and follow-up	66(30.3%)	67(30.7%)	31(14.2%)	164(75.2%)	54 (24.8)
Having access to professional counseling (e.g., psychologist, social worker, counsellor, nurse specialist) if you, family or friends need it	53(24.3%)	66(30.3%)	42(19.3%)	161(73.9%)	57 (26.1)
Being informed about cancer which is under control or diminishing (that is, remission)	59(27.1%)	74(33.9%)	39(17.9%)	172(78.9%)	46 (21.1)
Being given an explanation of those tests for which you would like the explanation	54(24.8%)	77(35.3%)	31(14.2%)	162(74.3)	56 (25.7)
Being given written information about the important aspects of your care	58(26.6%)	72(33.0%)	36(16.5%)	166(76.1%)	52 (23.9)
**Health system information supportive care need**				**163(74.78)**	**55(25.22)**
**Physical daily living domain**					
Work around the home	58(26.6%)	63(28.9%)	51(23.4%)	172(78.9%)	46 (21.1)
Lack of energy/tiredness/Fatigue	65(29.8%)	79(36.2%)	49(22.5%)	193(88.5)	25 (11.5)
Not being able to do the things you used to do	43(19.7%)	72(33.0%)	65(29.8%)	180(82.5%)	38 (17.5)
Pain	79(36.2%)	50(22.9%)	34(15.6%)	163(74.4%)	55 (25.6)
Feeling unwell most fo the time	62(28.4%)	68(31.2%)	58(26.6%)	188(86.2%)	30 (13.8)
**Physical daily living supportive care need**				**180(82.57)**	**38(17.43)**
**Patient care support domain**					
More choice about which cancer specialists you see	60(27.5%)	65(29.8%)	35(16.1%)	160(73.4%)	58 (26.6)
More choice about which hospital you attend	41(18.8%)	78(35.8%)	38(17.4%)	157(72%)	61 (28)
Reassurance by medical staff that the way you feel is normal	59(27.1%)	63(28.9%)	35(16.1%)	157(72.1%)	61 (27.9)
Hospital staff attending promptly to your physical needs	66(30.3%)	71(32.6%)	24(11.0%)	161(73.9%)	57 (26.1)
Hospital staff acknowledging, and showing sensitivity to your feelings and emotional needs	66(30.3%)	58(26.6%)	30(13.8%)	154(70.7%)	64 (29.9)
**Patient care support needs**				**158(72.48)**	**60(27.52)**
**Sexuality domain**					
Changes in your sexual relationships	41(18.8%)	66(30.3%)	28(12.8%)	135(61.9%)	83 (38.1)
Changes in sexual feelings	56(25.7%)	59(27.1%)	17(7.8%)	132(60.6%)	86 (39.4)
To be given information about sexual relationships	35(16.1%)	52(23.9%)	44(20.2%)	131(60.2)	87 (39.8)
**Sexuality supportive care needs**				**133(61.01)**	**85(38.99)**
**Supportive care needs in 5 domains of supportive care**					
Patients with some need**				216 (99.08%)	
Patients with no need*					2 (0.92%)

The category patients with no need* indicates that this issue is either not a problem for them or they do not need help with this issue, as the need has been satisfied by the patients themselves. (indicated by 1—no need, not applicable; 2—no need, satisfied;) The category patients with some need** indicates that this issue causes them concern or discomfort, and their need for additional help ranges from low and moderate to high. (Indicated by 3—low need; 4—moderate need; 5—high need). The frequency (n) and percentage (%) were used for the analysis.

### Domain-wise assessment of supportive care needs

The psychological domain came 1st rank and the sexuality domain came 5th rank of supportive care needs with mean and standard deviations 70.29; 18.84 and 46.06; 34.16, respectively. The overall supportive care needs for the patients were with minimum 16.25, maximum 89.73, mean 55.92 and standard deviation 16.30 (Refer to Table 3).

**Table 3:** Domain-wise assessment of supportive care needs (n = 218)

Domains of supportive care needs	Minimum (%)	Maximum(%)	Mean	SD
Psychological	2.50	100	70.2982	18.84
Physical daily living	0	100	63.25	23.15
Health system information	6.82	100	57.9024	21.11
Patient care support	0	100	56.4679	21.92
Sexuality	0	100	46.0627	34.16
**Total Score**	16.25	89.73	55.92	16.30

*The score is based on 100 scores; SD, Standard deviation. The frequency (n) and percentage (%), mean and SD were used for analysis.*

### Association between domains of supportive care needs and selected variables

There was an association between age, marital status, family type, occupation, relationship status, current sexual activity and supportive care needs (sexuality domain), whereas no association was observed between age, education, marital status, hospital type and supportive care needs (psychological, physical daily living and health system information domain). (Refer to Table 4)

**Table 4:** Association between domains of supportive care needs and selected variables (n = 218)

Domain	Psy		PD		HI		PS		Se	
**Variables**	No	Some	No	Some	No	Some	No	Some	No	Some
**Age**										
Up to 64	2(1.1)	174(98.9)	9(5.1)	167(94.9)	13(7.4)	163(92.6)	22(12.5)	154(87.5)	47(26.7)	129(73.3)
65 and above	0(0.0)	42(100)	0(0.0)	42(100)	3(7.1)	39(92.9)	5(11.9)	37(88.1)	25(59.5)	17(40.5)
p-value	1.000		0.212		1.000		1.000		**0.000**	
**Education**										
Illiterate	1(0.6)	174(99.4)	5(2.9)	170(97.1)	14(8.0)	161(92.0)	17(9.7)	158(90.3)	59(33.7)	116(66.3)
Literate	1(2.3)	42(97.7)	4(9.3)	39(90.7)	2(4.7)	41(95.3)	10(23.3)	33(76.7)	13(30.2)	30(69.8)
p-value	0.356		0.078		0.744		**0.035**		0.720	
**Marital status**										
With life partner	2(1.2)	162(98.8)	7(4.3)	157(95.7)	13(7.9)	151(92.1)	24(14.6)	140(85.4)	29(17.7)	135(82.3)
Without life partner	0(0.0)	54(100)	2(3.7)	52(96.3)	3(5.6)	51(94.4)	3(5.6)	51(94.4)	43(79.6)	11(20.4)
p-value	1.000		1.000		0.766		0.097		**0.000**	
**Economic status**										
Enough to sustain for one year	1(0.6)	164(99.4)	4(2.4)	161(97.6)	11(6.7)	154(93.3)	23(13.9)	142(86.1)	54(32.7)	111(67.3)
Not enough to sustain for one year	1(1.9)	52(98.1)	5(9.4)	48(90.6)	5(9.4)	48(90.6)	4(7.5)	49(92.5)	18(34.0)	35(66.0)
p-value	0.428		**0.040**		0.547		0.337		0.868	
**Family type**										
Nuclear	1(0.7)	137(99.3)	6(4.3)	132(95.7)	11(8.0)	127(92.0)	15(10.9)	123(89.1)	39(28.3)	99(71.7)
Joint	1(1.2)	79(98.8)	3(3.8)	77(96.2)	5(6.2)	75(93.8)	12(15.0)	68(85.0)	33(41.2)	47(58.8)
p-value	1.000		1.000		0.790		0.398		**0.054**	
**Occupation**										
Non House wife	0(0.0)	116(100)	3(2.6)	113(97.4)	3(2.6)	113(97.4)	10(8.6)	106(91.4)	31(26.7)	85(73.3)
Housewife	2(2)	100(98)	6(5.9)	96(94.1)	13(12.7)	89(87.3)	17(16.7)	85(83.3)	41(40.2)	61(59.8)
p-value	0.218		0.310		**0.007**		0.055		**0.043**	
**Hospital type**										
Government	0(0)	50(100)	0(0.0)	50(100)	1(2)	49(98)	2(4)	48(96)	15(30)	35(70)
Non -government	2(1.2)	166(98.8)	9(5.4)	159(94.6)	15(8.9)	153(91.1)	25(14.9)	143(85.1)	57(33.9)	111(66.1)
p-value	1.000		0.123		0.127		**0.049**		0.732	
**Relationship status**										
Not living together	0(0)	83(100)	4(4.8)	79(95.2)	5(6)	78(94)	4(4.8)	79(95.2)	45(54.2)	38(45.8)
living together	2(1.5)	133(98.5)	5(3.7)	130(96.3)	11(8.1)	124(91.9)	23(17)	112(83)	27(20)	108(80)
p-value	0.526		0.734		0.790		**0.010**		**0.000**	
**Currently sexually active**										
No	23(14.8)	132(85.2)	31(20)	124(80)	58(37.4)	97(62.6)	62(40)	93(60)	96(61.9)	59(38.1)
Yes	10(15.9)	53(84.1)	23(36.5)	40(63.5)	20(31.7)	43(68.3)	25(39.7)	38(60.3)	15(23.8)	48(76.2)
p-value	0.837		**0.015**		0.533		1.000		**0.000**	

*Psy: Psychological, PD: Physical daily living, HI: Health system information, PS: Patient care support, Se: Sexuality.*

*Fisher exact test: p-value less than 0.05 is significant association; applied for cell value less than 5, Chi-square test: p-value less than 0.05 is significant association; applied for cell value more than 5.*

### Multicollinearity test

The multicollinearity test was carried out among independent variables associated with domains of supportive care needs. The following table shows the collinearity (relationship) test using Cramer’s V test with the p-value. The test was confirmed at a 5% significance level and were rearranged for model construction. (Refer to Table 5)

**Table 5:** Multicollinearity test for the predictors

Variables	Test value (using Cramer’s V)	p-value
Economic status Vs currently sexually active	0.016	0.812
Education status Vs type of hospital	0.114	0.242
Education status Vs relationship status	0.080	0.237
Type of hospital Vs relationship status	0.089	0.423
Age Vs marital status	0.312	**0.000**
Age Vs Family type	0.227	**0.001**
Age Vs occupation	0.125	0.066
Age Vs relationship status	0.168	**0.013**
Age Vs currently sexually active	0.157	**0.020**
Marital status Vs family type	0.114	0.092
Marital status Vs occupation	0.006	0.933
Marital status Vs relationship status	0.688	**0.000**
Marital status Vs currently sexually active	0.342	**0.000**
Family type Vs occupation	0.046	0.494
Family type vs. currently sexually active	0.086	0.202
Occupation Vs currently sexually active	0.010	0.886
Relationship Vs currently sexually active	0.458	**0.000**

Vs: Versus; Cramer’s V test: p-value less than 0.05 is significant association

#### Factors affecting different domains of supportive care needs

Binary logistic regression analysis was performed among associated variables after getting the results of the multicollinearity test to assess the effect of factors on domains of supportive care needs. The variables associated with different domains of supportive care needs included binary logistic regression. The physical daily living domain was significant with the currently sexually active status with a p-value 0.012 (AOR = 2.30, 95%CI: 1.20–4.39). The health system information domain was significant with occupation with a p-value 0.007(AOR = 5.5, 95%CI: 1.52–19.90). The patient care support domain was significant with the type of hospital with a p-value 0.000 (AOR = 8.45, 95%CI: 3.18–22.43). The sexuality domain was significant with the occupation with a p-value 0.001 (AOR = 3.04, 95%CI: 0.62 − 0.17) and the currently sexually active status with a p-value 0.000 (AOR = 5.74, 95%CI: 2.74–10.60) (Refer to Table 6 ).

**Table 6:** Binary logistic regression of factors associated with different domains of supportive care needs among cervical cancer patients (n = 218)

Domains		Need for Care				
		No need	Some Need	COR (95% CI)	AOR (95%CI)	p-value
**Physical daily living**						
Economic status	Enough to sustain for one year	4 (2.4)	161 (96.6)	1	1	0.779
	Not enough to sustain for one year	5 (9.4)	48 (90.6)	4.19(1.08, 16.23)	1.11(0.54, 2.27)	
Currently sexually active	No	31(20)	124(80)	1	1	**0.012**
	Yes	23(36.5)	40(63.5)	2.30(1.20,4.39)	2.30(1.20,4.39)	
**Health system information**						
Occupation	Non-housewife	3(2.6)	113(97.4)	1	1	**0.007**
	Housewife	13(12.7)	89(87.3)	5.5(1.52,19.90)	5.5(1.52,19.90)	
**Patient care support**						
Education status	Illiterate	17(9.7)	158(90.3)	1	1	0.451
	Literate	10(23.3)	33(76.7)	2.82(1.18, 6.70)	1.31(0.65, 2.67)	
Type of hospital	Government	2(4)	48(96)	1	1	**0.000**
	Non -government	25(14.9)	143(85.1)	4.2(0.96, 18.38)	8.45(3.18, 22.43)	
Relationship status	Not living together	4(4.8)	79(95.2)	1	1	0.166
	living together	23(17)	112(83)	4.06(1.35, 12.19)	1.5(0.84, 2.80)	
**Sexuality**						
					)	
Family type	Nuclear	39(28.3)	99(71.7)	1	1	0.058
	Joint	33(41.3)	47(58.7)	1.78(1.05, 3.18)	1.67 (1.14, 0.31)	
Occupation	Non-housewife	31(26.7)	85(73.3)	1	1	**0.001**
	Housewife	41(40.2)	61(59.8)	1.84(1.04, 3.26)	3.04 (0.62, 0.17)	
		)				
Currently sexually active	No	96(61.9)	59(38.1)	5.21(2.68, 10.12)	5.47 (2.74, 10.60)	**0.000**
	Yes	15(23.8)	48(76.2)	1	1	

AOR: adjusted odds ratio; COR: crude odds ratio, CI: confidence interval

## Discussion

This is the first study related to the SCNs of CC patients in Nepal. The study discloses different types of unmet SCNs of CC patients in all the domains with the highest prevalence in the psychological domain. The findings of this study would be significant for further planning of CC and cancer care in Nepal.

As findings show, 99.08% of the respondents needed some form of SC. According to Fitch, Page, & Porter (2008), cancer has extensive physical, emotional, social, and financial consequences for affected individuals and their families, and the cancer patients were found to suffer from multiple problems like fear of death, disfigurement, pain, disability, infertility, dependency, abandonment, altered relationships, and financial hardship [14]. Likewise, another study reported that cancer patients commonly struggled with the emotional impact of illness and a lack of control over their lives and they needed emotional, spiritual, informational, and financial support during all phases of treatment [57]. Depression and perceived stress are reported to be common among cancer patients in Nepal [58].

Consistent with our findings, one study in Indonesia with gynecological cancer patients found two categories of cancer (cervical 64.7% and ovary 35.3%). Among them, 96.1% of the respondents sought some need for SC and 3.9% of the respondents did not seek any SC [29].

This study found that CC patients reported the greatest need for SC in the areas of the psychological domain (87.9%). This is because cancer patients most commonly suffered from psychological problems. The main cause of psychological problems can be attributed to cancer diagnoses, treatment methods, and their adverse effects on patients’ thoughts and feelings [59]. According to Wenzel et al., 2002, women diagnosed with cancer may suffer mostly from psychological problems due to changes in their body image, the emerging effects of the disease, prognoses of treatment, and feelings of loss as cited in a phenomenological study in Turkey [60]. CC patients had a higher prevalence of psychiatric symptoms, including depression (33–52%) than patients with other gynecological malignancies [61]. Anxiety and fatigue are commonly identified problems among cancer patients, and among them 11% were CC in a hospital-based prospective study Nepal [37]. This study found the patients’ concern about those who were close to them (94%) as the top priority need in the psychological domain. This finding is supported by the study in Indonesia among gynecological cancer patients who prioritized (89.5%) psychological support to resolve their worries about those close to them [29]. The same study revealed that the patients with gynecological cancer prioritized SCNs related to psychological, physical daily living and sexuality domains [29]. Our finding is also supported by the study conducted in Toronto among lung cancer patients the study found that the highest proportion of unmet needs was related to psychological and physical domains [56]. Another study in California among lung cancer patients reported a high level of supportive care need being necessary in physical daily living domains followed by psychological and health system informational domains. This finding is similar to that of a study with breast cancer patients in Iran which reported patient care support (57.71, mean) being the fourth priority and sexuality (49.39, mean) being the last priority supportive care need [17]. Our finding is also consistent with the finding of the study with breast cancer patients in a tertiary hospital in Malaysia, which found that the highest unmet SCNs were observed in the psychological domain (Mean 53.31; SD ± 21.79), followed by the physical domain (Mean 38.16; SD ± 27.15) [62]. In contrast with the finding of our study, SCNs related to the health system information domain were the first priority SCNs among CC patients in northeast Thailand [36]. The variations in priority and type of SCNs may be due to variations in culture and personal values, sociodemographic characteristics of participants, approaches in the calculation of needs, assessment methods, timing, and sample characteristics [63].

The present research found that sexuality-related SCNs were the least prioritized needs by the participants. According to Byun et al. 2006, this may be because patients with CC were less interested in sexual relations due to more vaginal sexual symptoms such as a shortened and tightened vagina, vaginal dryness, and dyspareunia, compared with their pretreatment situation, as cited in a hospital-based cross-sectional study in Seoul [33]. Higher levels of sexual distress were shown to be associated with higher levels of vaginal sexual symptoms, worry about sexual-related pain, relationship dissatisfaction, and body image concerns [33]. The majority of the participating patients (46.7%), aged between 50 and 59, were in menopause and their sexual desire and hormone gradually decreased, causing both physical and mental changes [33]. With the decrease in sex hormones and aging sexual desire decreases, the vagina narrows, and the lubricant in the vagina decreases leading to a loss in interest in intercourse [64] The decrease in sexual interest may also make patients less likely to require support to meet their sexual needs [33]. Our finding is consistent with the study conducted among CC patients in northeast Thailand: sexuality need was the least priority of the samples (x = 1.63, S.D. = 0.94) [36].

Our study found age, education, marital status, economic status, family type, occupation, hospital type and participation in current sexual activity as the associated variables for SCNs among patients. This study revealed that younger patients had a greater number of unmet needs than older ones. It could be explained that younger patients are completely dependent on their families even to fulfill their basic needs and SCNs are influenced by sociodemographic factors such as age, sex, marital status, and income level and clinical factors such as the location of cancer, stage of disease, and tumor size. Factors influencing SCNs must be identified to ensure adequate care for cancer patients [65]. This finding is supported by the study conducted at Princess Margaret Cancer Centre in Toronto, Ontario among lung cancer patients found that younger patients had a greater number of unmet needs and, in contrast to our findings, education was not associated with the need for SC [56]. The present study also revealed that treatment modalities with or without radiation are not the associated variables for SCNs among CC patients. A study conducted in Australia which found that treatment modalities (radiotherapy and surgery) were not associated variables for SCNs among cancer patients [23]. In contrast to this finding, treatment modalities of surgery [24, 66] was the associated variable for SCNs among cancer patients and, surgery, radiotherapy and chemotherapy were associated variables for SCNs among prostate cancer patients [67]. Sociodemographic and clinical factors (e.g. age, sex, marital status, income level; and clinical factors such as the location of cancer, stage of disease, and tumor size) influence the pattern of perceived and expressed SCNs of cancer patients [65]. Our study also found that factors affecting supporting care needs among CC patients are occupation, hospital type, marital status and participation in sexual activity. This finding is congruent with that of a study carried out in Australia which found marital status and current employment as the factors affecting SCNs among cancer patients [23]. In contrast to this finding, age, and treatment modalities were identified as predictors of SCNs in the sexuality domain among cancer patients in Australia [24].

There are some limitations of this study. First, it adopted convenience sampling which affects the generality of the findings. Second, the participants were asked about their perceived needs for SC in 34 items under five bounded domains (psychological, health system and information, physical daily living, patient care and support, and sexuality), the study, therefore, did not explore the economic, social supportive need and supportive needs of caregivers which are equally crucial to assess SCNs for CC patients. Third, since the inclusion criteria of the study include CC patients of any stage so the perceived need in different stages of the CC may be different like patient in late stage may have more perceived needs than those in initial stage of CC. This may cause bias.

Finally, more than half of the respondents were illiterate and needed assistance in completing the survey. Although research assistants were given training on collecting data from illiterate participants, the participants’ dependency on research assistants might have caused an information bias.

### Clinical implications

Although there are some limitations of this study, it has some important clinical implications for the management of SCNs of CC patients and other cancer patients. The results have a significant implication for the kinds of CC patients, targeting and provision of SC services for women with CC who are under treatment. Most of the Nepali CC women under treatment had a high level of SCNs on the psychological domain followed by the physical daily living domain. An effective psychological and physical care intervention plays an important role in fulfilling SCNs as per CC patients’ perceived needs. It may be beneficial to assess SCNs of all cancer patients as routine screening care by using the questionnaire used in this study and a comparison of SCNs can be carried out among various types of cancer patients and intervention can be planned as per the needs of different kinds of cancer patients.

## Conclusion

This study pinpoints that Nepali CC patients have several unmet needs in all domains of SCNs with the first priority of SCNs on the psychological domain. Assessment of the SCNs of Nepali women with CC highlighted significant points that should be addressed to improve the care provided. The type of hospital was identified as a factor affecting SCNs. The CC patients who were receiving treatment from government hospitals were more in need of SC than other CC patients who were receiving treatment from non-government hospitals. So, it is necessary to provide sufficient SC for CC patients during their treatment period. All cancer hospitals in Nepal could start assessing the perceived SCNs of individual CC patients as routine care in different phases of treatment. It would be beneficial to address their needs as per the individual patient’s perceived needs, which promotes curing and caring for CC patients. Healthcare providers are to be familiarized with priority SCNs of CC patients. Early recognition and timely addressing of the perceived needs may then help to manage different types of difficulties with health system resources. Intervention studies can be planned on the priority basis of perceived SCNs among CC patients.

## Data Availability

The datasets used and/or analyzed during the current study are available from the corresponding author on reasonable request.

## Abbreviations

CC: Cervical cancer
LMICs: Lower-middle-income countries
SCNS-SF34: Supportive Care Needs Survey Short Form
SCNS –SF34N: Supportive Care Needs Survey Short Form Nepali
SCNS-LF59: Supportive Care Needs Survey Long Form
SPSS: Statistical Package for Social Science
SD: Standard Deviation
NHRC: Nepal Health Research Council
ZZU: Zhengzhou University
AOR: Adjusted odd ratio
COR: Crude odd ratio
